# Supplementary material for: Unveiling the Bioactive Potential of the Invasive Jellyfish Phyllorhiza punctata Through Integrative Transcriptomic and Proteomic Analyses
Source: Biomolecules. 2025 Aug 4;15(8):1121. doi: 10.3390/biom15081121 (PMC12383608; doi:10.3390/biom15081121)
Supplement: Supplementary file 1 [file biomolecules-15-01121-s001.zip › Suplementary Material/FileS3/PEPFOLD4/XP_065059349_6569/clusters.html]

 PEP-FOLD clusters


Download archive of all models  here   

|  |  |  |
| --- | --- | --- |
| # | file | sOPEP |
| # cluster 1 - Size: 2 |  |  |
|  | XP\_065059349\_6569-model1 | -24.4987 |
|  | XP\_065059349\_6569-model1.1 | -22.6923 |
| # cluster 2 - Size: 3 |  |  |
|  | XP\_065059349\_6569-model2 | -23.706 |
|  | XP\_065059349\_6569-model2.1 | -22.0115 |
|  | XP\_065059349\_6569-model2.2 | -21.5362 |
| # cluster 3 - Size: 1 |  |  |
|  | XP\_065059349\_6569-model3 | -23.092 |
| # cluster 4 - Size: 2 |  |  |
|  | XP\_065059349\_6569-model4 | -22.5001 |
|  | XP\_065059349\_6569-model4.1 | -20.3784 |
| # cluster 5 - Size: 1 |  |  |
|  | XP\_065059349\_6569-model5 | -21.7435 |
| # cluster 6 - Size: 8 |  |  |
|  | XP\_065059349\_6569-model6 | -21.7431 |
|  | XP\_065059349\_6569-model6.1 | -20.9044 |
|  | XP\_065059349\_6569-model6.2 | -20.5928 |
|  | XP\_065059349\_6569-model6.3 | -20.5487 |
|  | XP\_065059349\_6569-model6.4 | -20.325 |
|  | XP\_065059349\_6569-model6.5 | -20.0861 |
|  | XP\_065059349\_6569-model6.6 | -19.9896 |
|  | XP\_065059349\_6569-model6.7 | -19.7007 |
| # cluster 7 - Size: 1 |  |  |
|  | XP\_065059349\_6569-model7 | -21.7227 |
| # cluster 8 - Size: 1 |  |  |
|  | XP\_065059349\_6569-model8 | -21.6599 |
| # cluster 9 - Size: 4 |  |  |
|  | XP\_065059349\_6569-model9 | -21.6234 |
|  | XP\_065059349\_6569-model9.1 | -21.3649 |
|  | XP\_065059349\_6569-model9.2 | -20.8001 |
|  | XP\_065059349\_6569-model9.3 | -20.6445 |
| # cluster 10 - Size: 2 |  |  |
|  | XP\_065059349\_6569-model10 | -21.2924 |
|  | XP\_065059349\_6569-model10.1 | -20.0294 |
| # cluster 11 - Size: 10 |  |  |
|  | XP\_065059349\_6569-model11 | -21.2851 |
|  | XP\_065059349\_6569-model11.1 | -21.2737 |
|  | XP\_065059349\_6569-model11.2 | -20.8335 |
|  | XP\_065059349\_6569-model11.3 | -20.6456 |
|  | XP\_065059349\_6569-model11.4 | -20.6226 |
|  | XP\_065059349\_6569-model11.5 | -20.5292 |
|  | XP\_065059349\_6569-model11.6 | -20.4584 |
|  | XP\_065059349\_6569-model11.7 | -20.2411 |
|  | XP\_065059349\_6569-model11.8 | -20.164 |
|  | XP\_065059349\_6569-model11.9 | -20.0013 |
| # cluster 12 - Size: 2 |  |  |
|  | XP\_065059349\_6569-model12 | -21.2439 |
|  | XP\_065059349\_6569-model12.1 | -20.5012 |
| # cluster 13 - Size: 4 |  |  |
|  | XP\_065059349\_6569-model13 | -20.8731 |
|  | XP\_065059349\_6569-model13.1 | -20.3484 |
|  | XP\_065059349\_6569-model13.2 | -20.1731 |
|  | XP\_065059349\_6569-model13.3 | -19.8327 |
| # cluster 14 - Size: 3 |  |  |
|  | XP\_065059349\_6569-model14 | -20.8582 |
|  | XP\_065059349\_6569-model14.1 | -19.8573 |
|  | XP\_065059349\_6569-model14.2 | -19.6955 |
| # cluster 15 - Size: 1 |  |  |
|  | XP\_065059349\_6569-model15 | -20.8415 |
| # cluster 16 - Size: 4 |  |  |
|  | XP\_065059349\_6569-model16 | -20.7794 |
|  | XP\_065059349\_6569-model16.1 | -20.6594 |
|  | XP\_065059349\_6569-model16.2 | -20.0377 |
|  | XP\_065059349\_6569-model16.3 | -19.8845 |
| # cluster 17 - Size: 6 |  |  |
|  | XP\_065059349\_6569-model17 | -20.7132 |
|  | XP\_065059349\_6569-model17.1 | -20.4056 |
|  | XP\_065059349\_6569-model17.2 | -20.2423 |
|  | XP\_065059349\_6569-model17.3 | -20.1305 |
|  | XP\_065059349\_6569-model17.4 | -20.101 |
|  | XP\_065059349\_6569-model17.5 | -19.7711 |
| # cluster 18 - Size: 2 |  |  |
|  | XP\_065059349\_6569-model18 | -20.707 |
|  | XP\_065059349\_6569-model18.1 | -20.3312 |
| # cluster 19 - Size: 1 |  |  |
|  | XP\_065059349\_6569-model19 | -20.6722 |
| # cluster 20 - Size: 4 |  |  |
|  | XP\_065059349\_6569-model20 | -20.6161 |
|  | XP\_065059349\_6569-model20.1 | -20.0948 |
|  | XP\_065059349\_6569-model20.2 | -19.8397 |
|  | XP\_065059349\_6569-model20.3 | -19.3924 |
| # cluster 21 - Size: 1 |  |  |
|  | XP\_065059349\_6569-model21 | -20.4421 |
| # cluster 22 - Size: 1 |  |  |
|  | XP\_065059349\_6569-model22 | -20.3681 |
| # cluster 23 - Size: 2 |  |  |
|  | XP\_065059349\_6569-model23 | -20.2798 |
|  | XP\_065059349\_6569-model23.1 | -20.1314 |
| # cluster 24 - Size: 1 |  |  |
|  | XP\_065059349\_6569-model24 | -20.15 |
| # cluster 25 - Size: 1 |  |  |
|  | XP\_065059349\_6569-model25 | -20.0296 |
| # cluster 26 - Size: 3 |  |  |
|  | XP\_065059349\_6569-model26 | -19.9669 |
|  | XP\_065059349\_6569-model26.1 | -19.5942 |
|  | XP\_065059349\_6569-model26.2 | -18.6811 |
| # cluster 27 - Size: 1 |  |  |
|  | XP\_065059349\_6569-model27 | -19.7688 |
| # cluster 28 - Size: 1 |  |  |
|  | XP\_065059349\_6569-model28 | -19.2769 |
| # cluster 29 - Size: 5 |  |  |
|  | XP\_065059349\_6569-model29 | -18.2913 |
|  | XP\_065059349\_6569-model29.1 | -18.2498 |
|  | XP\_065059349\_6569-model29.2 | -18.2323 |
|  | XP\_065059349\_6569-model29.3 | -18.0477 |
|  | XP\_065059349\_6569-model29.4 | -16.4582 |
| # cluster 30 - Size: 2 |  |  |
|  | XP\_065059349\_6569-model30 | -18.1004 |
|  | XP\_065059349\_6569-model30.1 | -17.4951 |
| # cluster 31 - Size: 2 |  |  |
|  | XP\_065059349\_6569-model31 | -17.7791 |
|  | XP\_065059349\_6569-model31.1 | -17.375 |
| # cluster 32 - Size: 2 |  |  |
|  | XP\_065059349\_6569-model32 | -17.4534 |
|  | XP\_065059349\_6569-model32.1 | -16.4625 |
| # cluster 33 - Size: 2 |  |  |
|  | XP\_065059349\_6569-model33 | -17.3828 |
|  | XP\_065059349\_6569-model33.1 | -16.7372 |
| # cluster 34 - Size: 3 |  |  |
|  | XP\_065059349\_6569-model34 | -17.317 |
|  | XP\_065059349\_6569-model34.1 | -17.202 |
|  | XP\_065059349\_6569-model34.2 | -16.7647 |
| # cluster 35 - Size: 6 |  |  |
|  | XP\_065059349\_6569-model35 | -17.255 |
|  | XP\_065059349\_6569-model35.1 | -17.0832 |
|  | XP\_065059349\_6569-model35.2 | -16.8211 |
|  | XP\_065059349\_6569-model35.3 | -16.7232 |
|  | XP\_065059349\_6569-model35.4 | -16.723 |
|  | XP\_065059349\_6569-model35.5 | -16.3315 |
| # cluster 36 - Size: 1 |  |  |
|  | XP\_065059349\_6569-model36 | -17.0479 |
| # cluster 37 - Size: 1 |  |  |
|  | XP\_065059349\_6569-model37 | -16.7146 |
| # cluster 38 - Size: 2 |  |  |
|  | XP\_065059349\_6569-model38 | -16.7039 |
|  | XP\_065059349\_6569-model38.1 | -16.6944 |
| # cluster 39 - Size: 1 |  |  |
|  | XP\_065059349\_6569-model39 | -16.449 |
|  |  |  |
